# Local Mucosal Flap for the Treatment of Gingival Defect After Gingival Fibromatosis Excision

**DOI:** 10.7759/cureus.12016

**Published:** 2020-12-10

**Authors:** Konstantinos S Papadopoulos, Georgia Pantazidou, Eleni Karagkouni, George Papadopoulos, Ioannis Papaioannou

**Affiliations:** 1 Otolaryngology - Head and Neck Surgery, General Hospital of Patras, Patras, GRC; 2 Orthopedics, General Hospital of Patras, Patras, GRC

**Keywords:** gingival enlargement, gingival fibromatosis, mucosal flap, defect, gingival reconstruction

## Abstract

Gingival fibromatosis (GF) is a rare condition of fibrous enlargement of the gingivae causing functional or aesthetic problems. We report a case of localized GF in a 51-year-old healthy male patient who presented in our department with localized gingival enlargement. We performed gingivectomy and restored the defect with a novel local transpositional mucosal flap with excellent functional and aesthetic results. This type of intervention is accompanied by short surgical time, provides predictable results, and should be considered in adult patients with large defects from sizable lesions.

## Introduction

Gingival fibromatosis (GF) is a benign condition characterized by localized or generalized fibrous enlargement of the gingivae. Usually, GF progresses slowly and does not extend beyond the mucogingival junction. It can be hereditary, syndrome-related, drug-induced, or due to inflammatory causes. GF can affect both children and adults, causing functional and aesthetic problems. Hereditary GF can be idiopathic or related to syndromes, such as Zimmermann-Laband syndrome or gingival fibromatosis with hypertrichosis [1]. Drug-induced GF is associated with phenytoin, cyclosporine, and nifedipine [2]. Differential diagnosis includes fibrous epulis, pyogenic granulomas, osteomas, or other neoplasms [3]. There are many procedures available for treating gingival defects, although most of them are suboptimal to achieve a satisfactory outcome in cases of sizable lesions and subsequently large defects.

## Case presentation

**Figure 1:**
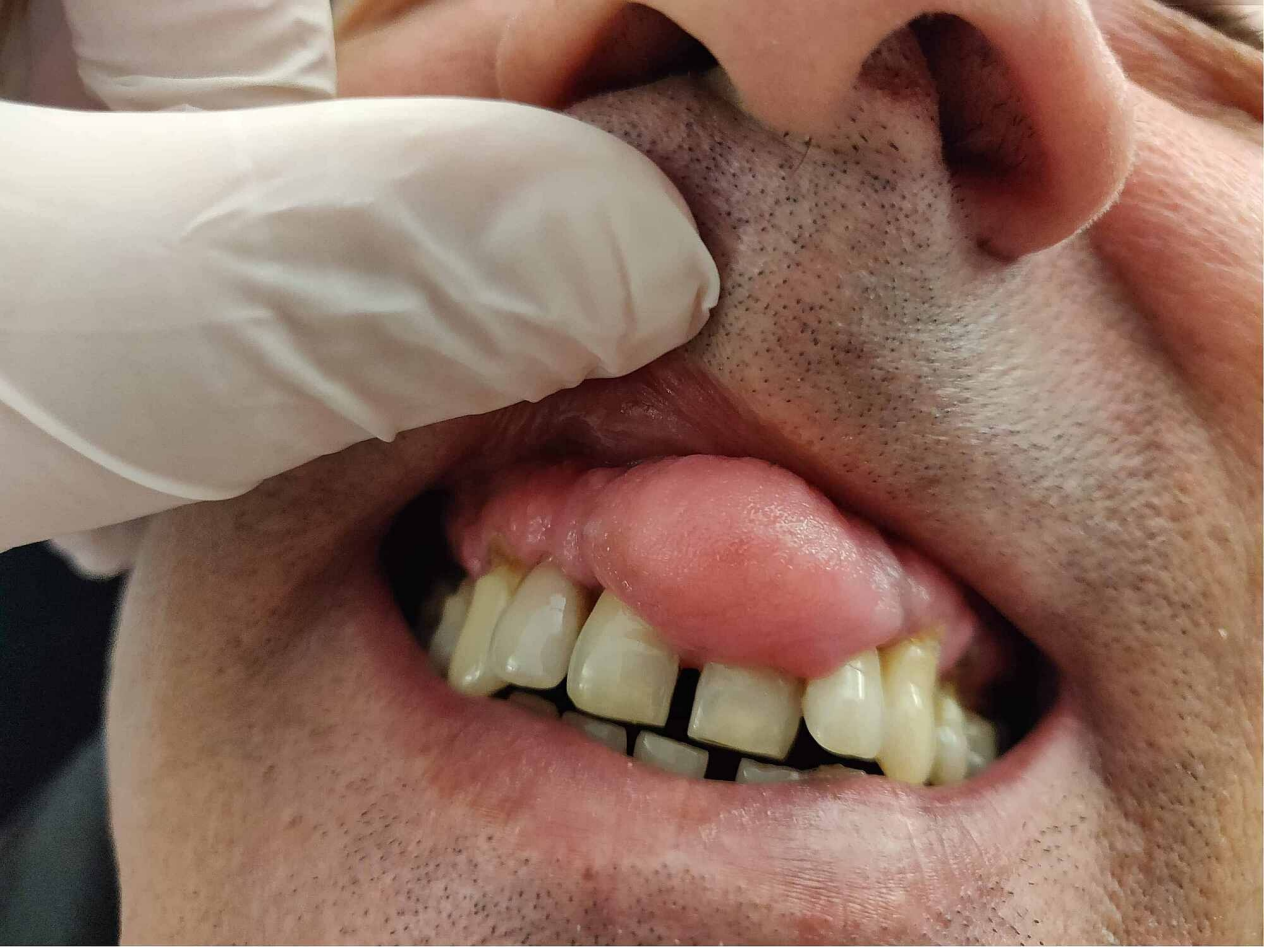
Preoperative image of the lesion

A 51-year-old male patient presented to our department with localized gingival overgrowth, which has progressed over four years (Figure 1).

**Figure 2:**
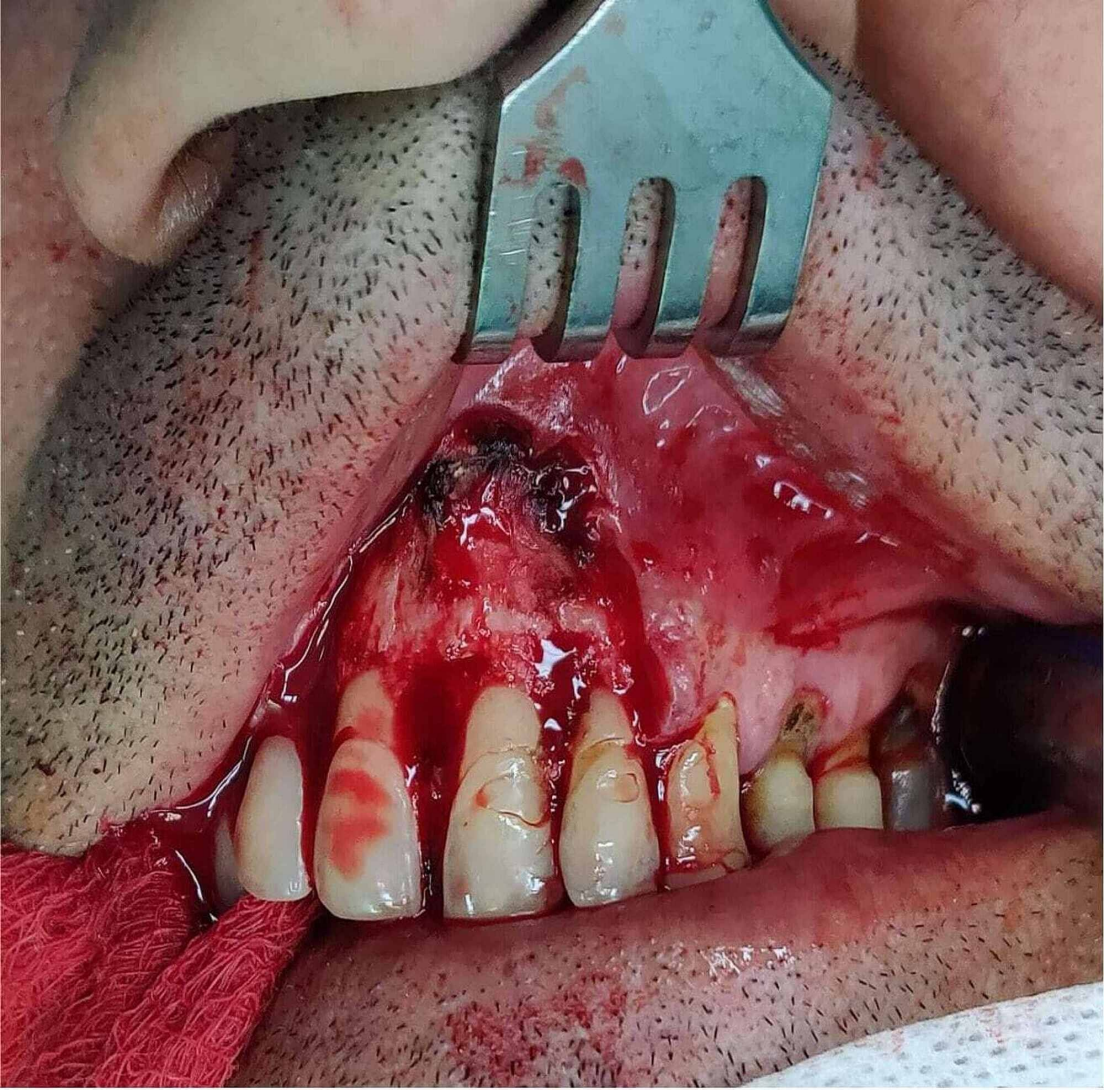
Gingival defect intra-operatively

The patient complained of minor compromise in upper lip functionality and principally for aesthetic discomfort. He had no comorbidities, although he was a social drinker and he had also smoked 20 cigarettes per day for the last 20 years. A simple excision was performed and the lesion was sent for biopsy. Histopathology examination confirmed the diagnosis of gingival fibromatosis. The size of the defect was approximately 3×2 cm (Figure 2).

**Figure 3:**
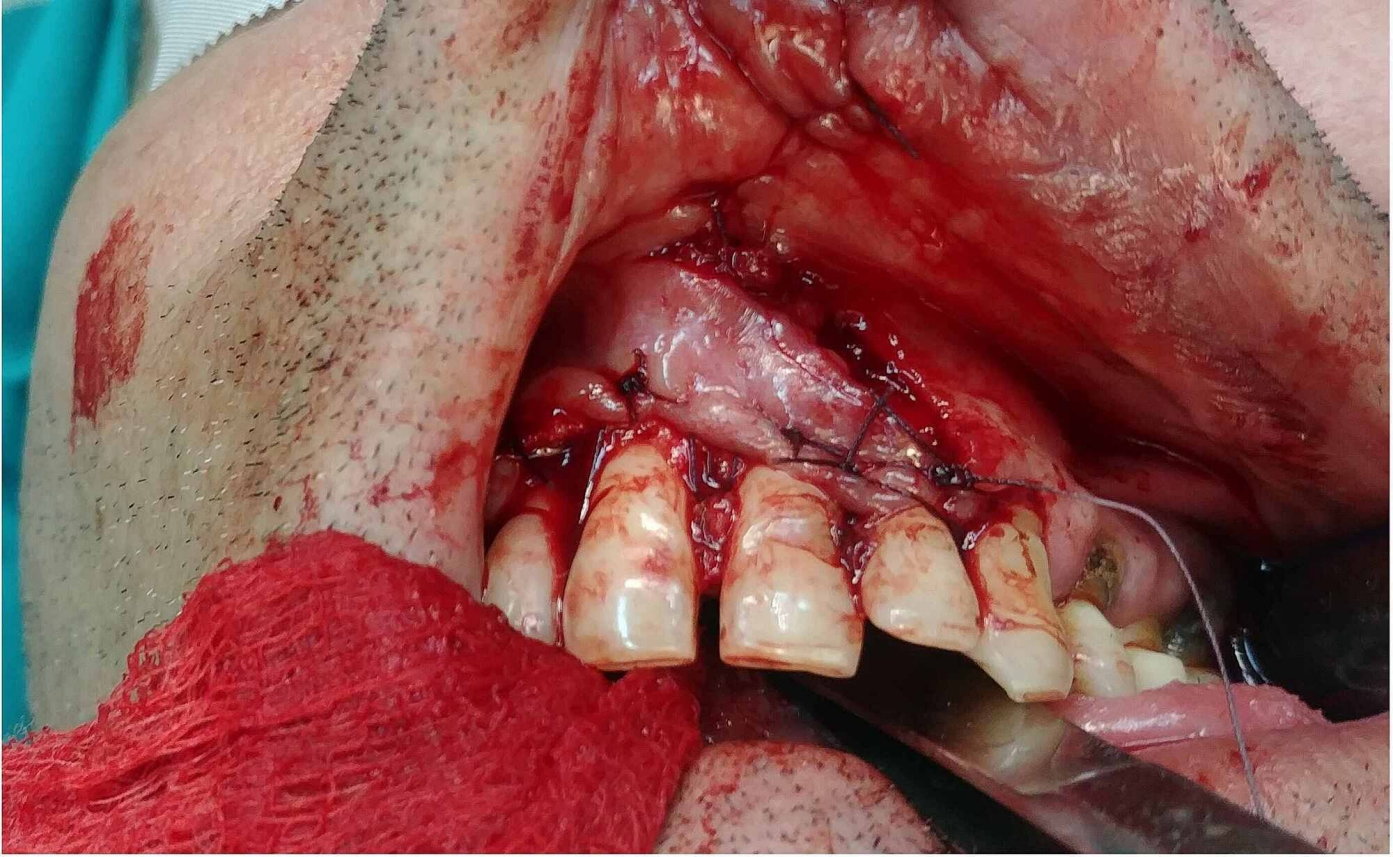
Flap suturing

A local triangular mucosal flap, with its pedicle at the mucogingival junction of the upper lip, was designed to cover the primary defect. The flap was raised to the level of the orbicularis oris muscle and was transpositioned and sutured in place of the gingival defect using 4-0 polyglycolic acid (PGA) sutures, while the secondary defect of the mucosa was sutured end-to-end with absorbable sutures (Figure 3). 

The patient had an uneventful recovery and was discharged the day after the surgery. No recurrence was observed during the 30-day follow-up, while the patient declared absolute satisfaction with the aesthetic, as well as the functional outcome.

## Discussion

Gingival defects from gum lesion excisions are troublesome and require precision to achieve aesthetically good results. Intraoral defects less than 4 cm are generally considered small, although the majority of the techniques (flap recovering) are too invasive [4]. Very small defects are traditionally treated with coronally advanced flaps with satisfactory outcomes, especially in pediatric patients [5]. Mucoperiosteal flaps have also been used in adults with GF, although the defect covering is insufficient in cases of large lesions excision [6-7].

Free connective tissue grafting is a very versatile option and can be combined with envelope techniques for stability and protection [8-9]. The advantages of grafts include the use of tissue with similar color and texture as the gingivae. This is of paramount importance when the defect occurs in the anterior region of the gums where a good aesthetic outcome is desirable. On the other hand, these flaps require considerable time to heal, while there is also the possibility of improper healing in cases with insufficient surrounding tissue [10]. The main advantage of the local mucosal flap is its vascularized pedicle. This ensures flap survival, as well as enhances the healing process. It can also be harvested anywhere across the mucogingival junction, depending on the location of the defect. The importance of flap management is based on the gentle tissue manipulation of the pedicle to avoid vascular compromise. Under these conditions, the procedure can be used with flexibility in gingival reconstruction. Transposition flaps for root coverage in cases with sufficient surrounding tissue have also been described as an alternative with better aesthetic results and higher survival rates compared to autografts [11].

Recurrence rates vary in the literature and the reported results are conflicting. High recurrence rates have been reported for hereditary GF cases in younger patients. This is probably due to the ongoing dentition process in these young patients. Delay in treatment in these subjects is absolutely reasonable [12].

A case series analysis showed that the gingivae with the alveolar ridge are the third most common site for oral squamous cell carcinomas [13], but the differential diagnosis is usually easy between benign and cancerous lesions. Despite that, intraoral masses should always be identified by biopsy or local excision in order to exclude malignancies masquerading as gingival overgrowth [14].

## Conclusions

Gingival fibromatosis is a rare and heterogeneous group of disorders that develop as slowly progressive, local or diffuse enlargements within marginal and attached gingiva or interdental papilla. The treatment consists of local excision and repair of the gingival margin. The size of the lesion plays a crucial role in the defect coverage. The local mucosal flap is a good alternative to traditional techniques, especially in cases with sizable lesions and defects. Surgeons should be aware of this technique since it is accompanied by exceptional functional and aesthetic results with a short healing time.
